# Quartz Crystal Microbalance Sensitivity Loss During Ionic Liquid Deposition: Insights into Film Structure and Morphology

**DOI:** 10.1002/cphc.70349

**Published:** 2026-04-10

**Authors:** Artur F. M. Farinha, Soraia R. M. R. Silva, Alexandre C. P. M. Alves, Luís M. N. B. F. Santos, Oleksandr Bondarchuk, José C. S. Costa

**Affiliations:** ^1^ CIQUP/Institute of Molecular Sciences (IMS) Departamento de Química e Bioquímica Faculdade de Ciências Universidade do Porto Porto Portugal; ^2^ International Iberian Nanotechnology Laboratory Braga Portugal; ^3^ SPIN‐Lab Centre for Microscopic Research on Matter University of Silesia in Katowice Chorzów Poland; ^4^ Institute of Chemistry University of Silesia in Katowice Katowice Poland

**Keywords:** film morphology and structure, ionic liquids, nanoscale confinement, quartz crystal microbalance (QCM), vapor deposition

## Abstract

The understanding of the sensitivity of quartz crystal microbalances (QCMs) to the mass of ionic liquid (IL) films is essential to rationalize their deposition behavior and the limits of reliable mass measurement. Herein, [C_2_C_1_im][OTf] films were vapor‐deposited on Au‐coated QCMs over a wide mass range (0.6–55 µg cm^−2^) and characterized using QCM, scanning electron microscopy (SEM), atomic force microscopy (AFM), and X‐ray photoelectron spectroscopy (XPS). At low coverages, QCM frequency shifts follow a linear trend, consistent with rigid mass coupling, while deviations from linearity and eventual sensor saturation occur at higher mass loadings. SEM and AFM show that the IL initially fills confined surface features, forming isolated patches, and progressively evolves into extended, quasi‐continuous domains. XPS indicates that low‐mass films display surface compositions deviating from stoichiometry, whereas high‐mass films (55 µg cm^−2^) show cation enrichment at the outermost surface. These findings demonstrate that QCM failure is linked to the transition from a confined, interface‐dominated regime to a microdroplet‐type, bulk‐like film, where viscoelastic IL patches decouple from the oscillating crystal. By correlating film morphology, surface composition, and QCM response, this study provides a comprehensive framework to interpret the limitations of QCM mass detection of IL films under vacuum.

## Graphical abstract

Vapor‐deposited ionic liquid films on Au‐coated QCMs evolve from confined, rigidly coupled layers to viscoelastic, bulk‐like overlayers as coverage increases. This transition explains the loss of QCM sensitivity and demonstrates how nanoscale confinement, substrate roughness, and IL properties govern reliable mass detection.

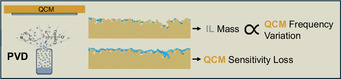

## 1 Introduction

Ionic liquids (ILs), salts that are liquid near ambient temperature, combine high thermal stability, negligible vapor pressure, and tunable interfacial and solvation properties, making them attractive media for thin‐film deposition and surface studies [1, 2, 3, 4, 5, 6, 7, 8]. Owing to their extremely low volatility, most ILs can be employed under high‐vacuum conditions, where conventional molecular liquids would rapidly evaporate or decompose [5, 6]. This exceptional vacuum stability provides a well‐defined platform for probing film growth, solvent–surface interactions, and the structure of liquids under nanoscale confinement while also enabling the use of ILs as functional solvents under high‐vacuum conditions [9, 10, 11, 12, 13, 14, 15, 16, 17, 18, 19, 20, 21]. The low volatility of ILs has enabled their direct investigation as vapor‐deposited thin films, which can exhibit morphologies ranging from isolated micro‐ and nanodroplets to continuous coalesced layers. Such systems serve as model platforms for examining interfacial tension, wetting behavior, and the influence of substrate chemistry on film organization [22, 23, 24, 25, 26, 27, 28, 29]. Our group has made extensive contributions to this field, specifically through the use of physical vapor deposition, demonstrating how IL molecular structure, substrate characteristics, and deposition parameters govern droplet formation and coalescence, as well as their evolution into flat, continuous films [30, 31, 32, 33, 34, 35, 36]. These insights into IL morphology and dynamics in vacuum have also paved the way for their use as functional templates in thin‐film growth. Building on pioneering work by Matsumoto and coworkers [11, 12, 13, 14, 15, 16], who demonstrated that IL layers can serve as solvent‐like media for the crystallization of organic semiconductors under vacuum, our studies helped to establish this so‐called vapor–liquid–solid (VLS) process [17, 18, 35].

To investigate such deposition processes in vacuum, the quartz crystal microbalance (QCM), typically coated with a thin gold layer, has become an invaluable tool. The QCM monitors shifts in the resonance frequency of a piezoelectric quartz crystal, which, under ideal conditions, is proportional to the mass adsorbed on its surface [37]. This relationship is described by the Sauerbrey equation, given in Equation (1) [38].

(1) $$\text{Δ}f=-\frac{2f^{2}}{\rho_{q}\cdot v_{q}\cdot A_{q}}\cdot \text{Δ}m_{q}=S_{q}\cdot \frac{\text{Δ}m_{q}}{A_{q}}$$

In this equation, *f* is the resonant frequency of the crystal, *ρ*_q_ is the density of the quartz, *v*_q_ is the shear acoustic wave velocity in quartz, and *A*_q_ is the effective area of the crystal. In this expression, *S*_q_ denotes the theoretical mass sensitivity coefficient, which can be derived from the intrinsic physical properties of the quartz crystal under the assumption of a homogeneous and rigid mass distribution over the active electrode area. For the 6 MHz AT‐cut quartz crystal employed as the resonator in the QCM used in this work, the theoretical mass sensitivity at *T* = 298 K is *S*_q_ = −81.5 Hz μg^−1^ cm^2^ [39]. During the effusion experiment, the temporal evolution of the quartz crystal resonance frequency directly reflects the accumulation of material on its surface. Under the assumptions of the Sauerbrey regime, the time derivative of the resonance frequency is proportional to the rate of mass uptake by the crystal and can be expressed according to Equation (2), where $\frac{\delta f}{\delta t}$ is the measured change in frequency over a finite time interval and $\frac{\delta m}{\delta t}$ is the corresponding mass deposition rate on the crystal. This relationship can be directly used to determine the deposition flux at the QCM surface. Defining the surface deposition rate, *φ*_dep_, as the mass deposited per unit area and per unit time, the flux can be expressed according to Equation (3). The *φ*_dep._ can be directly converted into a deposition rate in units of Å·s^−1^ by taking into account the density of the deposited material, providing an intuitive measure of film growth under vacuum conditions.

(2) $$\frac{\delta f}{\delta t}=S_{q}\cdot \frac{1}{A_{q}}\cdot \frac{\delta m}{\delta t}$$

(3) $$\varphi_{\mathrm{dep}.}=\frac{1}{A_{q}}\cdot \frac{\delta m}{\delta t}=\frac{1}{S_{q}}\cdot \frac{\delta f}{\delta t}$$

Owing to its high sensitivity and straightforward implementation, QCM is widely used to monitor vapor‐phase deposition processes, including those involving ILs [40, 41]. However, the quantitative application of QCM in these systems is complicated by the fact that the measured response often deviates from the ideal Sauerbrey relation, since IL films are neither rigid nor uniform and instead exhibit complex viscoelastic and morphological characteristics [41]. The oscillating crystal interacts dynamically with the liquid layer, and part of its vibrational energy is dissipated through viscous damping and molecular rearrangements within the film [42, 43, 44]. A characteristic manifestation of these effects is the saturation limit, an early deviation from the expected linear mass–frequency relationship that contrasts sharply with the behavior of solid films. In a study by Santos et al. on the vapor deposition of [C_2_C_1_im][NTf_2_] and [C_6_C_1_im][NTf_2_], the QCM response saturated at a frequency shift of only about Δ*f* ≈ 6 kHz, far below the values typically observed for solid films [41]. Interestingly, a semi‐crystalline IL, [C_14_C_1_im][NTf_2_], produced a higher saturation value (Δ*f* ≈ 15 kHz), suggesting a hybrid behavior in which partial solid‐like coupling coexists with liquid‐like mobility. These observations highlight that the apparent sensitivity loss is governed by the strength and nature of the interaction between the IL and the metal‐coated QCM surface: whether the molecules are rigidly coupled, as in a solid, or only loosely bound and able to slip, as in a liquid. Since IL morphologies are determined by substrate interactions, deposition rate, and surface diffusion, variations in these parameters can modulate both the structural organization and the viscoelastic response of the confined phase. To address these complexities, it is crucial to understand the deposition dynamics of ILs on the QCM surface, how they spread across the rough and fissured topography of the substrate, and whether they form large, droplet‐like domains resembling bulk liquid or thinner, more strongly coupled layers confined within surface irregularities. In this study, we use [C_2_C_1_im][OTf] as a model system to probe these behaviors.

## 2 Experimental Section

### 2.1 Materials and Methods

The IL 1‐ethyl‐3‐methylimidazolium triflate, [C_2_C_1_im][OTf], was acquired from IoLiTec with a stated purity of >99%. Detailed information on the IL, including its molecular structure and relevant physicochemical properties, is provided in the Supporting Information (Figure S1; Table S1). Prior to thin‐film deposition, residual volatile components were removed by thermal treatment under low pressure (*p* < 0.1 Pa) at 423 K. Thin films of [C_2_C_1_im][OTf] were deposited using an in‐house‐built Knudsen effusion apparatus [45]. The vacuum chamber was first evacuated from atmospheric pressure to approximately 1 Pa using a rotary pump, after which a diffusion pump further reduced the pressure to the 10^−4^ – 10^−3^ Pa range. Deposition was monitored in real time using a QCM integrated into the effusion chamber. Measurements were performed with 6 MHz gold‐coated sensor crystals (0.550” diameter) mounted in a commercial Inficon–Maxtek liquid‐cooled drawer single‐sensor head with a CDS‐A0F38 feedthrough. The resonance frequency of the quartz crystal was measured using a network analyzer in resonator measurement mode (Agilent E4915A). The crystal holder temperature was maintained at (278.2 ± 0.1) K. Although this differs from the temperature at which the reported Sauerbrey coefficient is given (298.15 K), the expected change in sensitivity over this range is well below the experimental uncertainty and does not affect the reported mass values. The deposited mass per unit area was calculated from the frequency shift of the quartz resonator, and deposition was continued until the QCM response exhibited a loss of sensitivity, marking the upper limit of detectable mass accumulation. Thin films were fabricated with the following deposited masses: (0.6 ± 0.1) µg cm^−2^, (1.2 ± 0.2) µg cm^−2^, (6 ± 1) µg cm^−2^, (26 ± 4) µg cm^−2^, and (55 ± 5) µg cm^−2^, corresponding approximately to IL thicknesses of (4 ± 1) nm, (9 ± 2) nm, (43 ± 7) nm, (187 ± 29) nm, and ≈ 400 nm, respectively. The latter value is indicative, as the QCM response had already saturated at this deposition.

### 2.2 Characterization Techniques

#### 2.2.1 Scanning Electron Microscopy

The surface morphology of the gold‐coated QCM crystals after IL deposition was examined by scanning electron microscopy (SEM) using a Hitachi FlexSEM 1000 instrument. Top‐view images were acquired at a magnification of 10 000× with a secondary electron (SE) detector, operating at an accelerating voltage of 3 kV and a working distance of 6 mm. All imaging parameters were carefully optimized to minimize possible electron‐beam‐induced effects on the IL films. Samples corresponding to [C_2_C_1_im][OTf] surface coverages of 0 (bare QCM), 0.6, 1.2, 6, 26, and 55 µg cm^−2^ were analyzed in order to compare different degrees of IL deposition and to assess the evolution of surface morphology with increasing deposited mass.

#### 2.2.2 Atomic Force Microscopy

Surface topography and roughness of the QCM crystals after deposition of [C_2_C_1_im][OTf] were characterized by atomic force microscopy (AFM) using a Bruker Dimension Icon system operated in tapping mode with a silicon cantilever (nominal resonance frequency ≈ 275 kHz). Images were acquired over scan areas of 5 × 5 µm at multiple locations on each sample to ensure representative surface characterization. AFM measurements were performed for IL surface coverages of 0, 0.6, 1.2, 6, and 26 µg cm^−2^. For each sample, multiple imaging channels (height, amplitude, and phase) were recorded to obtain complementary information on film morphology and local mechanical response. AFM data were processed and analyzed using the Gwyddion software package.

#### 2.2.3 X‐Ray Photoelectron Spectroscopy

X‐ray photoelectron spectroscopy (XPS) measurements were performed using an ESCALAB 250Xi spectrometer (Thermo Scientific) equipped with a monochromatic Al Kα X‐ray source (*hν* = 1486.6 eV, 200 W). The X‐ray beam spot on the sample surface was 650 µm. Spectra were collected at a 90° takeoff angle using a hemispherical electron energy analyzer operated in constant analyzer energy mode, with pass energies of 150 eV for survey spectra and 40 eV for high‐resolution spectra. The step size was 1.0 eV for survey scans and 0.1 eV for high‐resolution scans. High‐resolution spectra were recorded for the Au 4f, C 1s, N 1s, O 1s, F 1s, and S 2p regions. Data processing was performed using CasaXPS (version 2.3, Casa Software Ltd.), employing Gaussian–Lorentzian peak shapes and a Shirley‐type background [46]. Relative sensitivity factors were taken from the Kratos library. To assess lateral surface uniformity, XPS spectra were acquired at eight evenly spaced positions along a line across the QCM crystal surface for films with surface coverages of 26 and 55 µg cm^−2^.

## 3 Results and Discussion

### 3.1 QCM Response During IL Deposition

**FIGURE 1:**
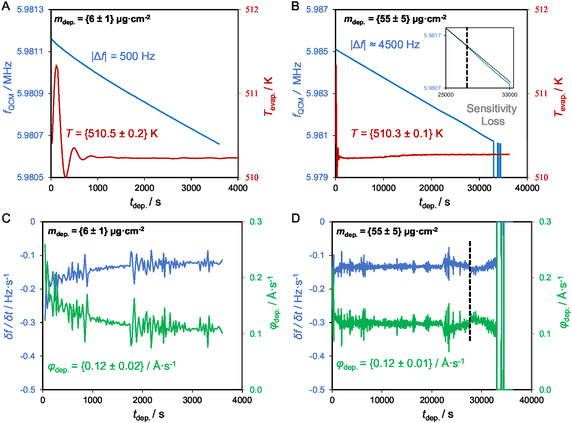
Plots showing the evolution of crystal frequency (*f*_QCM_) and evaporation temperature (*T*_evap._) as functions of deposition time (*t*_dep._) (graphs A and B), as well as the time derivative of the frequency (δ*f* / δ*t*) and the corresponding deposition rate (*φ*_dep._) (graphs C and D), for depositions of [C_2_C_1_im][OTf] onto Au‐coated quartz crystals. Two deposition amounts are shown: *m*_dep._ = {6 ± 1} and *m*_dep._ = {55 ± 5} µg cm^−2^. The dashed vertical lines in Figure 1B,D mark the boundary between the linear response region of the QCM and the onset of nonlinearity.

All depositions were carried out under highly reproducible conditions (Table 1) using freshly Au‐coated QCM substrates. Across the five independent experiments, the QCM frequency shifts (|Δ*f*|) ranged from 50 Hz to ≈ 4500 Hz, spanning nearly two orders of magnitude in deposited mass (*m*_dep._ = 0.6–55 µg cm^−2^) and film thickness (*l*_film_ = 4 – ≈ 400 nm). The nominal thickness (in nm) is presented only as an estimate, calculated from the deposited mass assuming the bulk density of the IL (Table S1) and that the deposited film is uniform and fully coalesced. The IL evaporation temperature (*T*_evap._) remained constant at 510.4 ± 0.2 K, well within the experimental uncertainty. Likewise, the real‐time deposition rate at the QCM surface (*φ*_dep._) was highly stable, with values tightly clustered around 0.12–0.13 Å s^−1^ for the majority of the deposition time. For all experiments, the initial flux was slightly higher (˜0.17 Å s^−1^) due to the clean substrate surface (effectively corresponding to a high sticking coefficient during the first instants of deposition). In the shortest deposition (|Δ*f*| = 50 Hz, *t* = 246 s), this resulted in a marginally higher measured flux. The QCM response exhibits very similar behavior for frequency shifts up to |Δ*f*| ≈ 4500 Hz. Beyond this threshold, however, a clear deviation in the QCM response is observed, indicating a change in the sensor performance at higher loadings. Figure 1 shows the evolution of the QCM frequency as a function of deposition time (*t*_dep._) for [C_2_C_1_im][OTf] films with two representative deposited masses, *m*_dep._ = 6 ± 1 and 55 ± 5 µg cm^−2^. The corresponding time derivatives of the frequency (δ*f* / δ*t*), together with the *φ*_dep_, are also shown, providing a clearer visualization of the QCM response during IL deposition. In addition, the temporal evolution of the evaporation temperature is included to demonstrate the thermal stability of the deposition conditions. Distinct deposition regimes are observed as a function of film thickness. As the evaporation temperature remains essentially constant throughout all experiments, a stable molecular flux arriving at the surface is implied. Under these conditions, the QCM frequency is expected to decrease linearly with time as long as the deposited film remains rigidly coupled to the crystal. This behavior is well reproduced for |Δ*f*| values of 50, 100, 500, and 2000 Hz (Figure 1A,C illustrate this behavior for |Δ*f*| = 500 Hz). For the thickest deposition, however, the frequency evolution departs from linearity slightly before the ultimate failure of the crystal. Despite the unchanged evaporation temperature, the QCM response progressively deviates from linear behavior, ultimately leading to saturation and failure, as shown in Figure 1B,D. This behavior is clearly reflected in the time‐derivative plots, where δ*f* / δ*t* becomes progressively less negative, indicating a reduction in the apparent deposition rate despite a steady incoming molecular flux. These features indicate that the QCM begins to saturate prior to complete failure. Rather than indicating a reduction in the arrival rate of IL molecules, the diminished frequency response reflects the progressively reduced ability of the QCM to transduce additional deposited mass into proportional frequency shifts. The onset of QCM saturation is therefore clearly identified at |Δ*f*| ≈ 4500 Hz, corresponding to a deposited mass of ≈ 55 µg cm^−2^. This onset was consistently reproduced across multiple independent deposition experiments, indicating that the sensitivity loss is an intrinsic and reproducible feature of the IL/QCM system. A plausible interpretation of this behavior is that, at low deposited masses, the IL preferentially occupies confined or nanoscopic features of the QCM. As the deposited mass increases, these confined sites become saturated, and the IL progressively forms a quasi‐continuous layer on the Au/QCM surface. In this regime, the film can no longer follow the oscillatory motion of the quartz crystal purely elastically, resulting in partial mechanical decoupling, increased dissipation, and ultimately the breakdown of the Sauerbrey approximation. The onset of this transition may coincide with the observed deviation from linearity and precedes the ultimate failure of the crystal at the highest loadings.

**TABLE 1:** Experimental data for the deposition of [C_2_C_1_im][OTf] onto Au‐coated quartz crystals. Parameters include evaporation temperature (*T*_evap._), substrate temperature (*T*_subst._), variation of frequency of the Au‐coated quartz crystal after each IL deposition (|Δ*f*|_QCM_), deposition rate at the QCM surface (*φ*_dep._), deposition time (*t*_dep._), mass deposited per unit area (*m*_dep._), and film thickness (*l*_film_).

IL	*T*_evap._	*T*_subst._	|Δ*f*|_QCM_	*φ*_dep._	*t*_dep._	*m*_dep._	*l*_film_
	K	K	Hz	Å s^−1^	s	µg cm^−2^	nm
[C_2_C_1_im][OTf]	510.6 ± 0.5	278.2 ± 0.1	50	0.17 ± 0.03	246	0.6 ± 0.1	4 ± 1
	510.3 ± 0.3		100	0.12 ± 0.02	749	1.2 ± 0.2	9 ± 2
	510.5 ± 0.2		500	0.12 ± 0.02	3575	6 ± 1	43 ± 7
	510.3 ± 0.1		2000	0.13 ± 0.02	14 365	26 ± 4	187 ± 29
	510.3 ± 0.1		≈ 4500	0.12 ± 0.01	32 962	55 ± 5	≈ 400

### 3.2 Evolution of Surface Morphology

**FIGURE 2:**
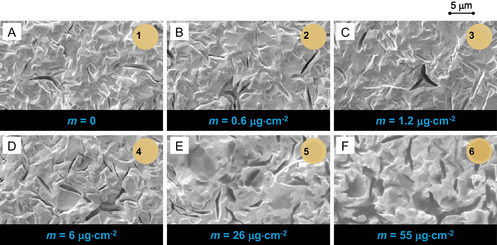
SEM top‐view images (10,000× magnification) of Au‐coated quartz crystals after the deposition of 0 (A), 0.6 (B), 1.2 (C), 6 (D), 26 (E), and 55 µg cm^−2^ (F) of [C_2_C_1_im][OTf]. Images were acquired using the SE detector. Insets 1–6 show corresponding photographs of the crystal surfaces taken without magnification (naked‐eye view). All IL depositions were performed under identical conditions: a substrate temperature of 278.15 K, an evaporation temperature of 510 K, and a deposition rate of 0.1 Å s^−1^.

**FIGURE 3:**
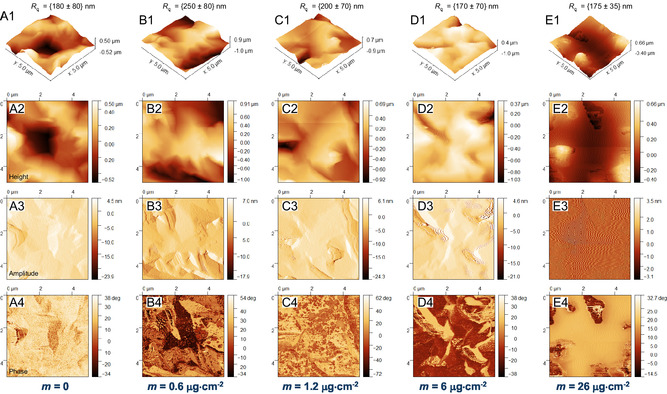
AFM images of the Au‐coated quartz crystal surface after deposition of [C_2_C_1_im][OTf] at surface coverages of 0 (A1–A4), 0.6 (B1–B4), 1.2 (C1–C4), 6 (D1–D4), and 26 (E1–E4) µg cm^−2^. Each row corresponds to a different imaging channel: height (first and second rows), amplitude (third row), and phase (fourth row). Images were acquired in tapping mode with a scan size of 5 × 5 µm.

Figure 2 presents SEM micrographs of Au‐coated quartz crystals after IL deposition at increasing film thicknesses. The evolution of surface morphology with increasing IL coverage provides direct visual evidence of film organization. At low coverages (0.6–6.0 µg cm^−2^), SEM imaging reveals no discernible IL features (Figure 2B–D). The surface appears identical to the bare QCM (Figure 2A), indicating that the deposited material remains confined within the irregularities of the substrate and does not yet form detectable surface features. Clear morphological signatures of the IL emerge only at higher coverages (26 and 55 µg cm^−2^, Figure 2E,F). At 26 µg cm^−2^, IL is visible primarily inside cracks and depressions, forming localized pools that follow the underlying roughness. In contrast, at 55 µg cm^−2^, extended and connected IL regions cover substantial areas of the surface. This transition is significant, as the formation of these large, coalesced domains provides a plausible explanation for the loss of QCM sensitivity observed at high mass loadings. Once the film evolves into a continuous layer, viscous IL regions with weak mechanical coupling to the substrate are no longer fully locked to the crystal's resonance frequency, leading to viscoelastic decoupling, increased dissipation, and instability in resonance tracking. AFM imaging with multiple detection channels (height, amplitude, and phase) provides complementary information on both the topography and mechanical properties of the deposited films (Figure 3). For the highest deposited mass (55 µg cm^−2^), AFM imaging was not feasible, as the formation of a thick, soft IL layer led to unstable tip–sample interactions and rapid tip contamination, precluding reliable topographic measurements. In the height maps (rows 1 and 2), no discernible IL features are observed for coverages up to 6 µg cm^−2^ (images B1, B2, C1, C2, D1, D2); the morphology is indistinguishable from the pristine rough surface of the Au/QCM (images A1, A2), consistent with the expectation that small amounts of IL are confined and are not individually resolved at this scale. Clear IL domains only appear at 26 µg cm^−2^ (images E1, E2), matching the threshold at which SEM images also begin to reveal visible material. This observation naturally connects to the QCM response shown earlier. The AFM‐measured roughness of the substrate (≈170–250 nm) is close to the IL thickness at which the QCM begins to lose quantitative accuracy in Figure 1 (≈ 400 nm). This indicates that the IL initially settles into the surface depressions and only forms domains on the surface once its thickness matches the typical height of those roughness features. The amplitude images (row 3), which reflect the local slope of the surface, show the characteristic contrast of the rough QCM for low coverages, whereas the 26 µg cm^−2^ sample exhibits a nearly featureless dark image. This indicates that the cantilever is interacting with a mechanically more homogeneous and dissipative surface, consistent with the presence of viscous IL patches. The phase images (row 4) provide the highest contrast between surfaces with different mechanical responses. For low deposited amounts, the phase signal mirrors the exposed QCM topography. From 6 µg cm^−2^ onwards, darker regions appear in the cracks, indicating a more dissipative response from IL confined in these depressions. In the 26 µg cm^−2^ sample, bright domains dominate the image, corresponding to IL‐rich areas, while isolated dark “islands” remain where the substrate is still exposed, typically corresponding to topographical highs.

Interestingly, even some depressions remain uncovered, illustrating that once thick coalesced IL regions form, they preferentially spread laterally rather than spontaneously filling nearby deeper features. This behavior is consistent with the viscoelastic nature of IL domains, which have a limited ability to flow into small or narrow features. These four channels provide a coherent picture: IL is initially confined to the surface roughness, becomes detectable as isolated patches only at higher coverages, and eventually forms continuous, dissipative domains that substantially modify the mechanical response sensed by the AFM probe.

### 3.3 Chemical Composition and Surface Coverage

**FIGURE 4:**
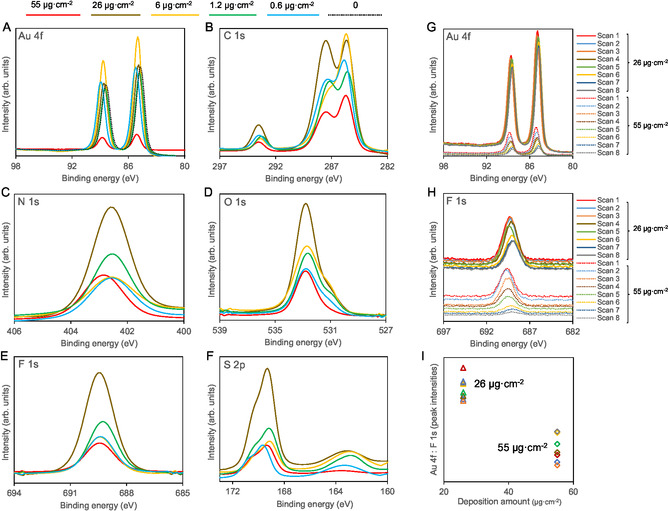
High‐resolution XPS spectra of [C_2_C_1_im][OTf] film samples at surface coverages of 0 (dashed black curve), 0.6 (solid blue curve), 1.2 (solid green curve), 6 (solid yellow curve), 26 (solid brown curve), and 55 (solid red curve) µg cm^−2^, deposited on Au‐coated quartz crystal surfaces. The spectra were acquired for Au 4f (graphs A and G), C 1s (graphs B), N 1s (graph C), O 1s (graph D), F 1s (graphs E and H), and S 2p (graph F). The data presented in graphs G and H were acquired from eight scans taken along a line across the surface. Graph I shows the ratio between the maximum intensities of the Au 4f and F 1s core‐level peaks, based on the data presented in graphs G and H.

Figure 4 shows the high‐resolution XPS spectra of [C_2_C_1_im][OTf] films at different surface coverages. XPS survey spectra are presented in the Supporting Information (Figures S2–S7). XPS analysis confirms the progressive coverage of the Au substrate by IL and provides insights into the chemical uniformity of the films. Although minor variations are observed at intermediate coverages, the Au 4f signal (Figure 4A) undergoes pronounced attenuation with increasing IL deposition and reaches its lowest intensity at 55 µg cm^−2^, consistent with near‐complete surface coverage. This observation agrees with the SEM and AFM results, which indicate that, at this deposition level, the Au/QCM surface is predominantly covered by the IL. The IL‐specific core‐level signals (F 1s, S 2p, N 1s, and O 1s) are most intense for the 26 µg cm^−2^ sample, indicating that at this coverage a significant fraction of the substrate is already coated by IL, as also observed in the SEM and AFM images. The maximum XPS core‐level peak intensities as a function of the deposition amount are presented in Figure S8. For the thickest sample (55 µg cm^−2^), all peaks show a relative decrease in intensity compared to the intermediate coverages. This might be related to the IL's different morphology or molecular arrangement at high coverage, becoming more “bulk‐like” and less firmly bound to the substrate. The spatial homogeneity of the thicker films was assessed through XPS measurements acquired at eight evenly spaced positions across the QCM surface. The Au 4f and F 1s spectra (Figure 4G,H) reflect the relative exposure of the Au substrate and IL coverage, respectively. As expected, the 55 µg cm^−2^ sample exhibits a weaker Au signal and a stronger fluorine signal than the 26 µg cm^−2^ film, confirming that the higher coverage yields more extensive surface coverage. Across the scanned positions, slight variations in peak intensity are observed for both Au and fluorine, suggesting minor lateral heterogeneity of the films (Figure 4I). Interestingly, both signals exhibit a slight decrease in intensity upon consecutive scans. Although the underlying mechanism cannot be unambiguously identified, this behavior may be associated with subtle modifications of the IL surface induced by repeated X‐ray exposure or other experimental factors.

**FIGURE 5:**
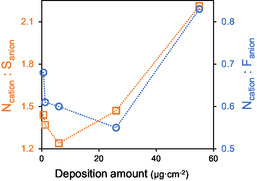
Ratios of N 1s / S 2p (N_cation_/S_anion_, open squares) and N 1s/F 1s (N_cation_/F_anion_, open circles) core‐level XPS peak intensities as a function of deposition amount for [C_2_C_1_im][OTf] films. The theoretical values assuming a 1:1 stoichiometry between [C_2_C_1_im] and [OTf] are N/F ≈ 0.667 and N/S = 2. Measured values correspond to deposited masses of 0.6, 1.2, 6, 26, and 55 µg cm^−2^ (see details in Table S2).

To further support the discussion, the surface composition of the IL films was evaluated by analyzing the N 1s / F 1s and N 1s / S 2p XPS intensity ratios. Since nitrogen is exclusively associated with the imidazolium cation, whereas fluorine and sulfur are solely present in the triflate anion, these ratios provide a qualitative measure of the relative cation‐to‐anion abundance at the film surface. Assuming a 1:1 stoichiometry between [C_2_C_1_im] and [OTf], the theoretical ratios are N/F = 2/3 ≈ 0.667 and N/S = 2/1 = 2. The measured N/F ratios were 0.68, 0.61, 0.60, 0.55, and 0.83, while the corresponding N/S ratios were 1.44, 1.37, 1.24, 1.47, and 2.21. These values correspond to increasing deposited masses of 0.6, 1.2, 6, 26, and 55 µg cm^−2^, respectively (Table S2). To better visualize these trends, Figure 5 plots the obtained ratios as a function of deposition amount. The structure of the IL layer is expected to differ depending on whether it is deposited on flat surfaces or within confined regions. For instance, Maier et al. showed that [C_2_C_1_im][OTf] forms a homogeneous submonolayer wetting layer on Au, with cations and anions arranged in a checkerboard pattern, upon which subsequent deposition leads to pronounced 3D growth [47]. Deposition on the Au/QCM surface is further influenced by confinement within nanoscale surface features, which affects both the IL organization and the exposure of ionic species at the outermost surface. For deposited masses up to 26 µg cm^−2^, the measured N/F and N/S ratios are slightly below the theoretical values. This indicates a slight deviation from the stoichiometric composition at the outermost surface of the films, likely due to the confinement of the IL within the nanoscopic features of the Au‐coated quartz crystal. Such confinement can lead to an asymmetric distribution of the ionic species near the interface. In this regime, the reduced relative nitrogen signal may arise either from a preferential interaction of one of the ionic species with the Au substrate or from geometric effects associated with confinement, where part of the imidazolium cation is less exposed to the vacuum. An exception is observed for the smallest deposition (0.6 µg cm^−2^), where the N/F ratio is close to the theoretical value. The relative deviation of this film from the other samples is small and likely falls within the typical uncertainty of quantitative XPS analysis. Nevertheless, both N/F and N/S ratios are higher than those obtained for the 1.2 and 6 µg cm^−2^ samples, which may reflect slight differences in local ordering, surface interactions, or other experimental factors.

At the highest deposited mass (55 µg cm^−2^), the N/F and N/S ratios increase above the theoretical values, coinciding with the formation of extended IL domains and with the onset of QCM saturation. This reflects a transition from a confined interfacial regime to a less confined, more bulk‐like film morphology. However, the exact molecular origin of the surface composition cannot be uniquely determined from the present data. The observed differences in surface composition between the confined and bulk phases might be understood as a consequence of mainly thermodynamic factors, with interfacial interactions playing a dominant role. In the confined phase, the interactions between the IL and the solid surface become dominant, altering the natural anion–cation arrangement and thereby influencing the measured N/F ratio by XPS. This surface‐induced ordering is a thermodynamically driven effect, as the system minimizes its free energy by adopting a configuration that accommodates the interfacial forces. In contrast, at higher deposited quantities (55 µg cm^−2^), a bulk‐like IL phase is formed. In this regime, the influence of the substrate is screened, and the ions adopt a more random orientation. Taken together, these observations indicate that the film deposited at 55 µg cm^−2^ exhibits a surface composition that is clearly distinct from that of thinner films (≤26 µg cm^−2^), consistent with a transition from a confined interfacial regime to a less confined, more extended IL layer that increasingly defines the outermost surface. Although the present data do not allow the exact molecular origin of this surface composition to be uniquely determined, the results clearly demonstrate a change in both morphology and surface chemistry as the confined sites become saturated. These results align with previous reports, which show that confined ILs can adopt morphologies and surface compositions distinct from those of bulk‐like films, where molecular mobility and exposure at the outermost surface increase as the coverage increases [48, 49, 50, 51, 52, 53, 54, 55].

### 3.4 Mechanism of QCM Sensitivity Loss

**FIGURE 6:**
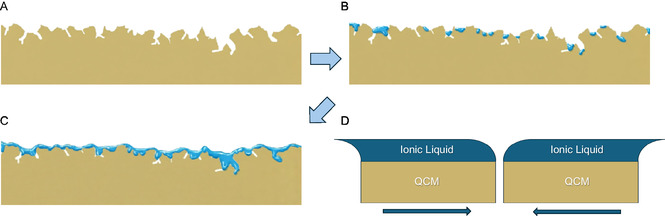
Schematic cross‐section of IL deposition progression on a QCM. (A) Bare gold‐coated surface with inherent surface roughness. (B) At low coverage, IL is confined within topographic features, forming isolated droplets in craters and valleys. (C) At high coverage, IL droplets coalesce into a continuous, viscoelastic overlayer that bridges the surface features. Arrows indicate the sequence of IL deposition. The transition from (B) to (C) corresponds to the onset of mechanical decoupling and QCM sensitivity loss. (D) Schematic of interfacial slip and viscous lag in a thick IL film during QCM shear oscillation. The same Au‐coated QCM substrate (yellow) is shown at the two extremes of its lateral motion, coated by a continuous IL film (blue). In both positions, the QCM motion is only partly transmitted to the IL, which undergoes interfacial slip and internal shear deformation, resulting in mechanical decoupling and viscous energy dissipation, making the QCM response insensitive to further increases in IL mass.

The combined evidence from QCM monitoring, surface imaging, and chemical analysis reveals a clear progression in IL organization that directly explains the observed saturation in mass sensitivity. This mechanism is driven by a transition from a rigidly coupled, confined film to a mechanically decoupled, viscoelastic overlayer, as illustrated schematically in Figure 6. Under the constant evaporation flux maintained throughout these experiments (Table 1), the Sauerbrey equation predicts a linear decrease in QCM frequency with deposited mass. This ideal behavior is observed at low surface coverages, where the frequency decreases linearly until the target mass is reached. At this stage, the deposited [C_2_C_1_im][OTf] is confined within the topography of the Au‐coated substrate. SEM and AFM imaging reveal no distinct morphological features of IL; the surface appears identical to that of the bare substrate. The IL molecules fill nanoscale cracks, depressions, and irregularities, remaining in intimate contact with the gold. This initial state of topographic confinement, where the IL is fully sequestered within surface features, is depicted in Figure 6B. In this configuration, the IL is spatially constrained and moves rigidly with the oscillating crystal, thereby fulfilling the Sauerbrey condition and enabling accurate mass sensing. As deposition continues, the IL begins to form distinct domains that extend above the surface roughness. A clear morphological transition occurs: IL‐specific domains become visible in SEM and AFM, evolving from localized pools within cracks to extended, interconnected regions. XPS data confirms this, with IL signals maximizing at 26 µg cm^−2^ as the Au 4f signal attenuates. This process is schematically illustrated in the progression shown in Figure 6, where confined droplets coalesce into a continuous overlayer that bridges the substrate's topographic features. Some deeper zones may remain unfilled due to the IL's viscosity. However, it is clear that a bulk‐like film forms across the surface. This transition from confinement to overlayer formation fundamentally alters the mechanical interaction with the QCM. The newly formed continuous IL domains are no longer constrained by the substrate topography and exhibit inherent viscoelasticity. As shown in Figure 6C,D, this results in mechanical decoupling: while the quartz crystal oscillates rapidly, the thick, viscous IL layer cannot follow this motion rigidly. It experiences interfacial slip and significant internal viscous dissipation. Consequently, a portion of the deposited mass is dynamically “invisible” to the sensor. The onset of sensitivity saturation (≈55 µg cm^−2^, corresponding to a nominal thickness of ≈400 nm) is close to the substrate's characteristic roughness (≈170–250 nm). This correlation confirms that the IL must first fill the available topographic features before forming the decoupled, bulk‐like domains responsible for the loss of QCM sensitivity. The maximum mass that a QCM can reliably measure clearly depends on the specific IL, reflecting differences in molecular size, viscosity, and the ability to occupy confined surface regions before forming a mechanically decoupled overlayer. For instance, [C_2_C_1_im][NTf_2_] and [C_6_C_1_im][NTf_2_] films reach QCM saturation at frequency shifts of ≈ 6000 Hz [41], whereas [C_2_C_1_im][OTf] saturates at ≈ 4500 Hz. This difference may arise from competing effects of molecular size and transport properties. In the case of [C_2_C_1_im][OTf], its higher viscosity compared to the [NTf_2_]‐based congeners could limit its ability to rapidly fill confined regions, promoting earlier viscoelastic decoupling. For thin IL films, viscosity and diffusivity may often dominate the QCM response, although surface affinity can also play an important role. The specific effect of molecular size is not straightforward, as it may vary between ILs. Taken together, these results demonstrate that QCM sensitivity loss is an inherent consequence of the IL's transition from a rigidly confined film to a viscoelastic overlayer and that both the IL type and substrate topography critically influence the maximum measurable mass. Based on our proposed mechanism, several practical strategies may help mitigate QCM sensitivity loss during IL vacuum deposition. Users should first determine, for each specific IL–QCM combination, the mass loading at which deviations from Sauerbrey linearity appear, since this threshold depends on viscosity, wetting behavior, and surface morphology. Adjusting the QCM surface (e.g., changing electrode material, roughness, or crystal supplier) can influence IL penetration into confined regions and thus shift the saturation threshold. Moreover, deliberately introducing a rough or porous underlayer prior to IL deposition may increase the available nanoscale confinement volume, delaying the onset of bulk‐like film formation and extending the Sauerbrey‐valid regime to higher deposited masses.

## 4 Conclusion

The study demonstrates that the deposition behavior of [C_2_C_1_im][OTf] on Au‐coated QCMs is strongly influenced by surface confinement, film morphology, and substrate topography. At low deposited masses, the IL remains confined within nanoscale surface features, forming isolated patches and exhibiting deviations from stoichiometric surface composition. When deposition reaches 55 µg cm^−2^ (≈ 400 nm), the [C_2_C_1_im][OTf] film reorganizes into extended domains that increasingly occupy the outermost surface, leading to mechanical decoupling and QCM saturation. Depending on the IL, the extent of QCM saturation is likely governed by the IL's ability to occupy confined regions rather than adsorb on the surface, with substrate roughness playing a key role. Combined morphological and chemical analyses demonstrate how nanoscale confinement dictates both film structure and mechanical response, providing critical insight into the limitations of mass detection for soft, viscoelastic IL films under vacuum. These findings are crucial for accurately interpreting QCM measurements, optimizing vapor‐deposition protocols, and informing the design of IL‐based thin films and coatings in functional applications, such as electronics, catalysis, and surface functionalization.

## Supporting Information

Additional supporting information can be found online in the Supporting Information section. **Supporting Fig. S1**: Molecular structure of 1‐ethyl‐3‐methylimidazolium triflate, [C_2_C_1_im][OTf]. **Supporting Fig. S2**: XPS survey spectrum of the Au‐coated QCM substrate, confirming the presence of Au together with minor contributions from adventitious carbon and oxygen. **Supporting Fig. S3**: XPS survey spectrum of the [C_2_C_1_im][OTf] film deposited on the Au/QCM substrate at a surface coverage of 0.6 µg cm^−2^ showing characteristic Au 4f, C 1s, N 1s, O 1s, F 1s, and S 2p signals. **Supporting Fig. S4**: XPS survey spectrum of the [C_2_C_1_im][OTf] film deposited on the Au/QCM substrate at a surface coverage of 1.2 µg cm^−2^ showing characteristic Au 4f, C 1s, N 1s, O 1s, F 1s, and S 2p signals. **Supporting Fig. S5**: XPS survey spectrum of the [C2C1im][OTf] film deposited on the Au/QCM substrate at a surface coverage of 6 µg cm^−2^ showing characteristic Au 4f, C 1s, N 1s, O 1s, F 1s, and S 2p signals. **Supporting Fig. S7**: XPS survey spectrum of the [C_2_C_1_im][OTf] film deposited on the Au/QCM substrate at a surface coverage of 55 µg cm^−2^ showing characteristic Au 4f, C 1s, N 1s, O 1s, F 1s, and S 2p signals. **Supporting Fig. S8**: Maximum XPS core‐level peak intensities (Au 4f, N 1s, O 1s, F 1s, and S 2p) as a function of the deposition amount. **Supporting Table S1**: CAS registry number (CAS), molar mass (M), density (ρ), viscosity (η), melting temperature (T_m_), and superficial tension (γ) values for the ionic liquid [C_2_C_1_im][OTf]. **Supporting Table S2**: Experimental N_cation_ : F_anion_ and N_cation_ : S_anion_ ratios derived from the XPS data.

## Conflicts of Interest

The authors declare no conflicts of interest.

## Supporting information

Supplementary Material

## Data Availability

The data that support the findings of this study are available from the corresponding author upon reasonable request.
