# Supplementary material for: Quartz Crystal Microbalance Sensitivity Loss During Ionic Liquid Deposition: Insights into Film Structure and Morphology
Source: Chemphyschem. 2026 Apr 10;27(7):e70349. doi: 10.1002/cphc.70349 (PMC13068631; doi:10.1002/cphc.70349)
Supplement: Supplementary file 1 — Supplementary Material [file CPHC-27-e70349-s001.pdf]

Supporting Information (SI)

QCM Sensitivity Loss During Ionic Liquid Deposition: Insights into Film Structure and Morphology

Artur F. M. Farinha,[a] Soraia R. M. R. Silva,[a] Alexandre C. P. M. Alves,[a] Luís M. N. B. F. Santos,[a] Oleksandr Bondarchuk,[b] and José C. S. Costa[a]*

[a] CIQUP/Institute of Molecular Sciences (IMS), Departamento de Química e Bioquímica, Faculdade de Ciências, Universidade do Porto, Rua do Campo Alegre s/n, 4169–007 Porto, Portugal.

[b] International Iberian Nanotechnology Laboratory, Av. Mestre José Veiga, s/n, 4715-330, Braga, Portugal; SPIN-Lab Centre for Microscopic Research on Matter, University of Silesia in Katowice, 75 Pułku Piechoty Str. 1A, Chorzów 41-500, Poland; Institute of Chemistry, University of Silesia in Katowice, 9 Szkolna Str., 40-006 Katowice, Poland.

* Corresponding author.

E-mail: jose.costa@fc.up.pt

The Supporting Information provides relevant details on the ionic liquid [C_2C_1im][OTf], as well as XPS characterization data for the IL films deposited on the Au/QCM substrate at different surface coverages.

[C_2C_1im][OTf]

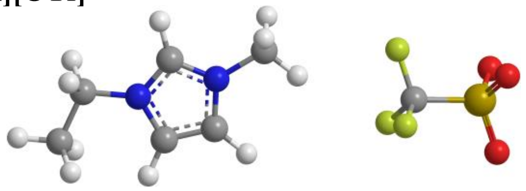

Figure S1. Molecular structure of 1-ethyl-3-methylimidazolium triflate, [C_2C_1im][OTf].

Table S1. CAS registry number (CAS), molar mass (M), density (ρ), viscosity (η), melting temperature (T_m), and superficial tension (γ) values for the ionic liquid [C_2C_1im][OTf].

Ionic Liquid	CAS	M / $g \cdot mol^{-1}$	ρ (298K) / $g \cdot cm^{-3}$	η / mPa·s	T_m / K	γ / $mN \cdot m^{-1}$
[C_2C_1im][OTf]	145022-44-2	260.23	1.39[1]	45.7[2] (298K)	258[3]	41.3[4] (298K)<br>39.2[5] (298K)

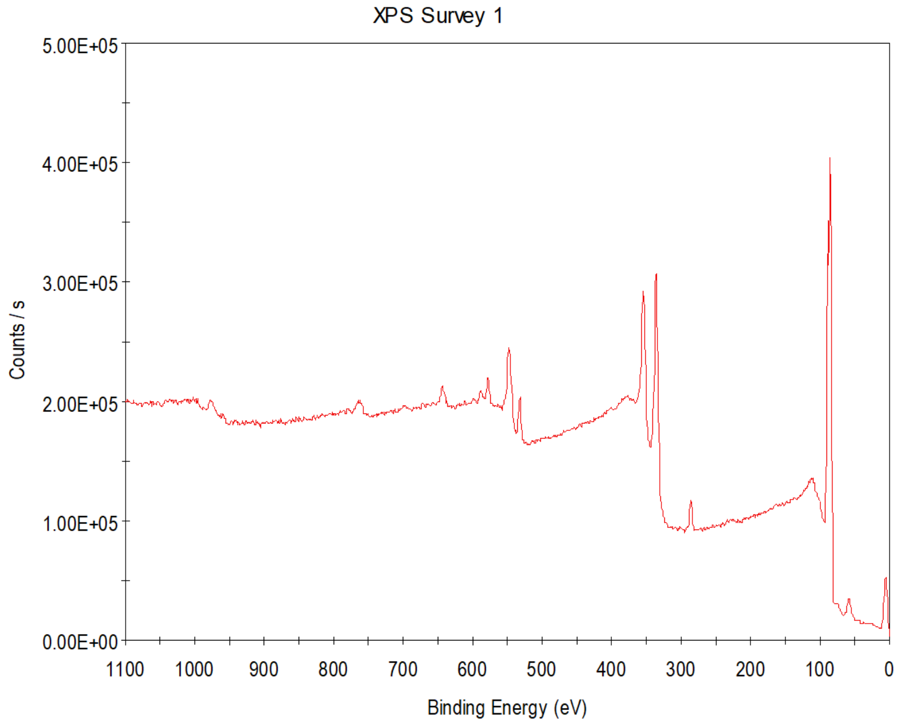

Figure S2. XPS survey spectrum of the Au-coated QCM substrate, confirming the presence of Au together with minor contributions from adventitious carbon and oxygen.

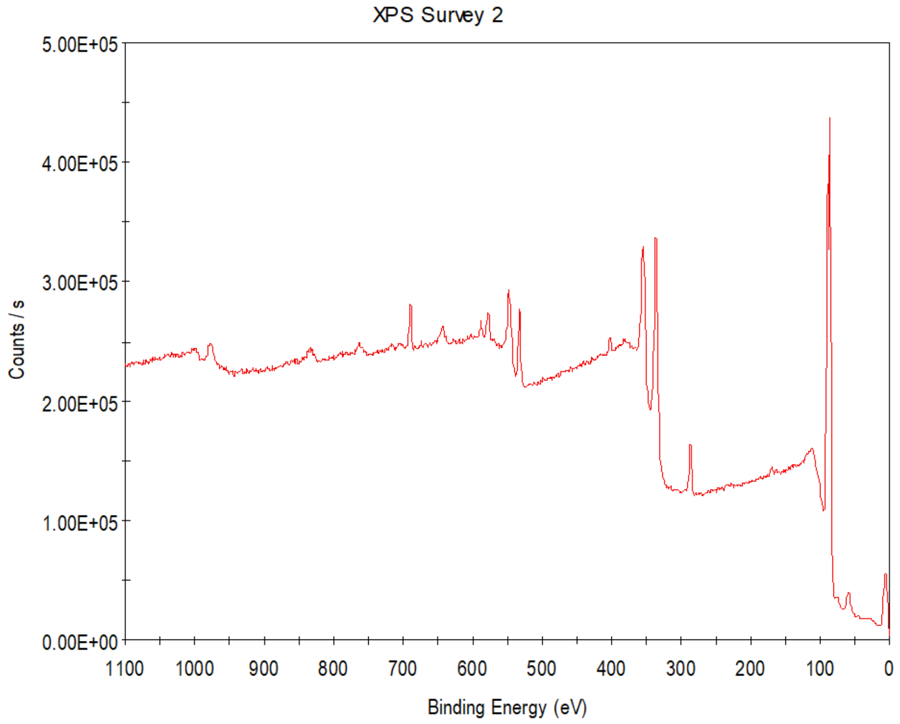

Figure S3. XPS survey spectrum of the $[C_2C_1im][OTf]$ film deposited on the Au/QCM substrate at a surface coverage of 0.6 $\mu g \cdot cm^{-2}$ showing characteristic Au 4f, C 1s, N 1s, O 1s, F 1s, and S 2p signals.

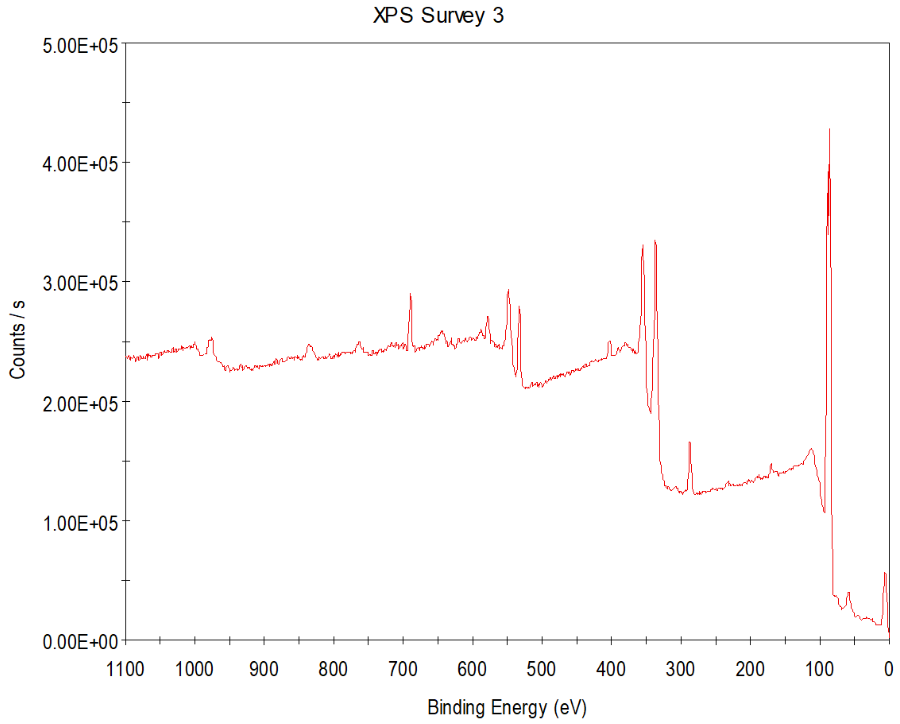

Figure S4. XPS survey spectrum of the [C_2C_1im][OTf] film deposited on the Au/QCM substrate at a surface coverage of 1.2 μg·cm^{-2} showing characteristic Au 4f, C 1s, N 1s, O 1s, F 1s, and S 2p signals.

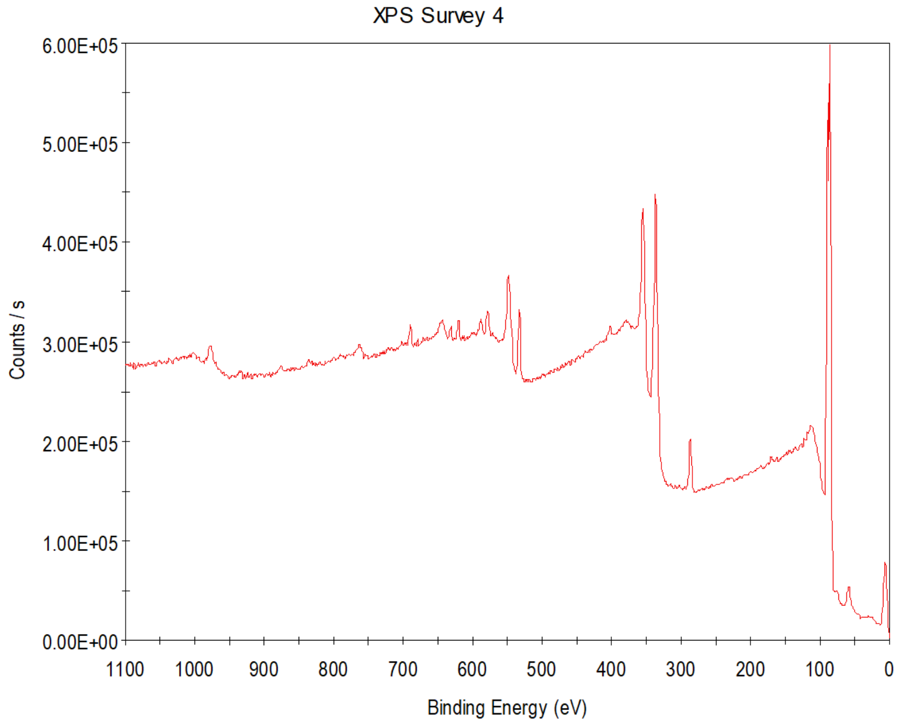

Figure S5. XPS survey spectrum of the [C_2C_1im][OTf] film deposited on the Au/QCM substrate at a surface coverage of 6 μg·cm^{-2} showing characteristic Au 4f, C 1s, N 1s, O 1s, F 1s, and S 2p signals.

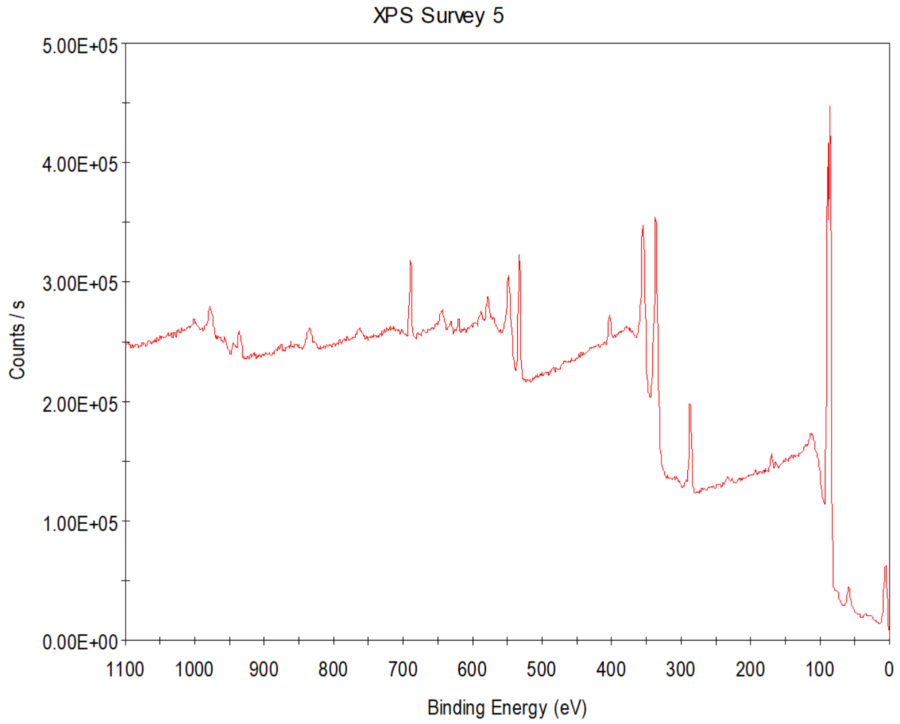

Figure S6. XPS survey spectrum of the $[C_2C_1im][OTf]$ film deposited on the Au/QCM substrate at a surface coverage of 26 µg·cm^{-2} showing characteristic Au 4f, C 1s, N 1s, O 1s, F 1s, and S 2p signals.

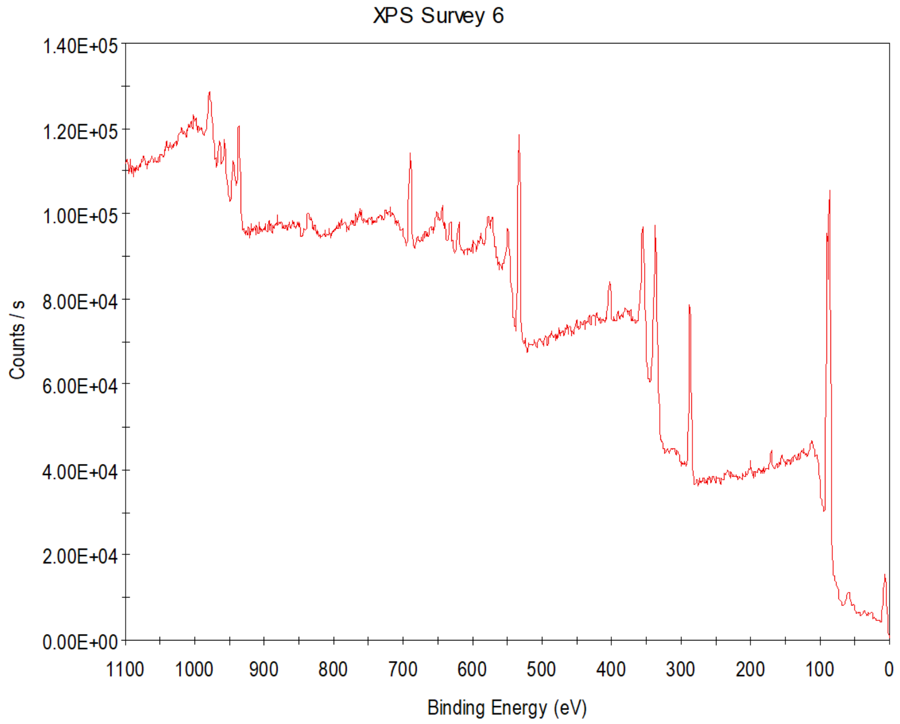

Figure S7. XPS survey spectrum of the $[C_2C_1im][OTf]$ film deposited on the Au/QCM substrate at a surface coverage of 55 µg·cm^{-2} showing characteristic Au 4f, C 1s, N 1s, O 1s, F 1s, and S 2p signals.

XPS core-level intensities as a function of the IL deposition amount

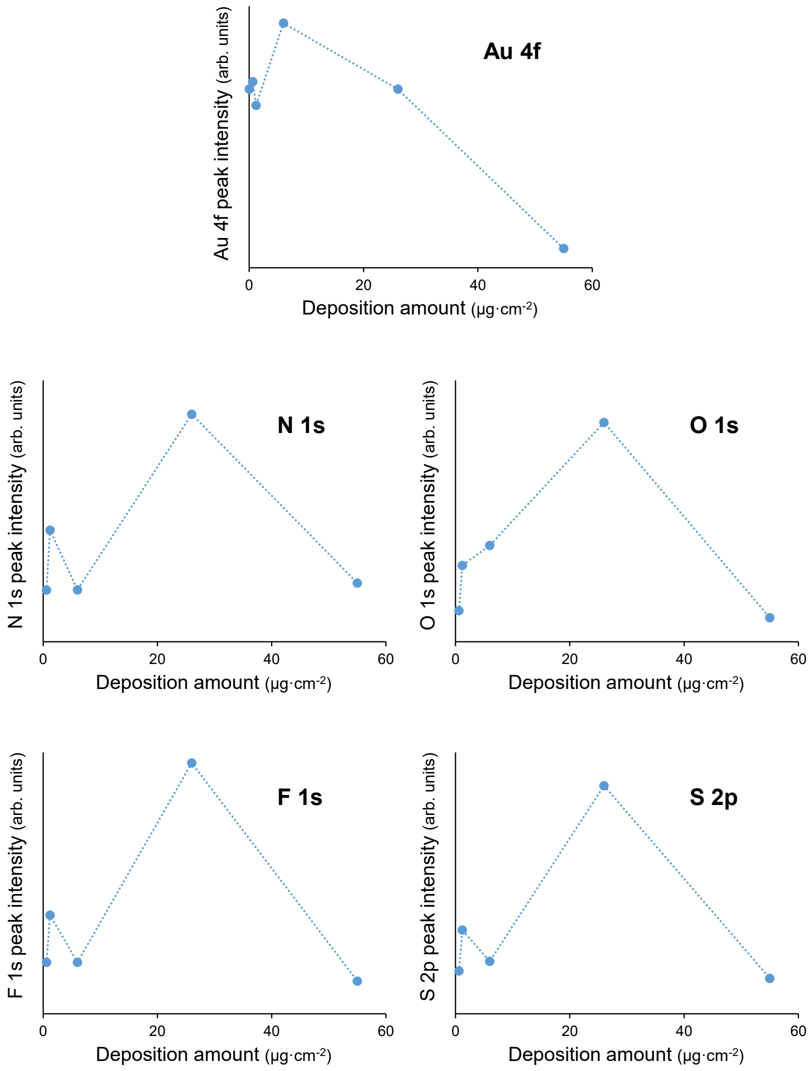

Figure S8. Maximum XPS core-level peak intensities (Au 4f, N 1s, O 1s, F 1s, and S 2p) as a function of the deposition amount.

Table S2. Experimental N_{cation} : F_{anion} and N_{cation} : S_{anion} ratios derived from the XPS data.

Sample	N_{cation} / F_{anion}	N_{cation} / S_{anion}
substrate: Au-coated quartz crystal surface		
**$[C_2C_1im][OTf]$ (0.6 μg·cm^{-2})**	0.68	1.44
**$[C_2C_1im][OTf]$ (1.2 μg·cm^{-2})**	0.61	1.37
**$[C_2C_1im][OTf]$ (6 μg·cm^{-2})**	0.60	1.24
**$[C_2C_1im][OTf]$ (26 μg·cm^{-2})**	0.55	1.47
**$[C_2C_1im][OTf]$ (55 μg·cm^{-2})**	0.83	2.21
Expected (stoichiometric)	0.67	2

References

(1) J. Klomfar, M. Součková, J. Pátek, *J. Chem. Eng. Data* **2010**, *55*, 4054.
(2) N. Anwar, Riyazuddeen. *J. Chem. Eng. Data* **2018**, *63*, 269.
(3) H. Every, A. G. Bishop, M. Forsyth, D. R. MacFarlane, *Electrochim. Acta* **2000**, *45*, 1279.
(4) H. F. D. Almeida, A. R. R. Teles, J. A. Lopes-da-Silva, M. F. Freire, J. A. P. Coutinho, *J. Chem. Thermodyn.* **2012**, *54*, 49.
(5) M. Tariq, M. G. Freire, B. Saramago, J. A. P. Coutinho, J. N. C. Lopes, L. P. N. Rebelo, *Chem. Soc. Rev.* **2012**, *41*, 829.
